# Supplementary material for: GROOLS: reactive graph reasoning for genome annotation through biological processes
Source: BMC Bioinformatics. 2018 Apr 11;19:132. doi: 10.1186/s12859-018-2126-1 (PMC5896057; doi:10.1186/s12859-018-2126-1)

# Observations to concepts

Concept

Is it predicted?

Is it expected?

# Observations to concepts

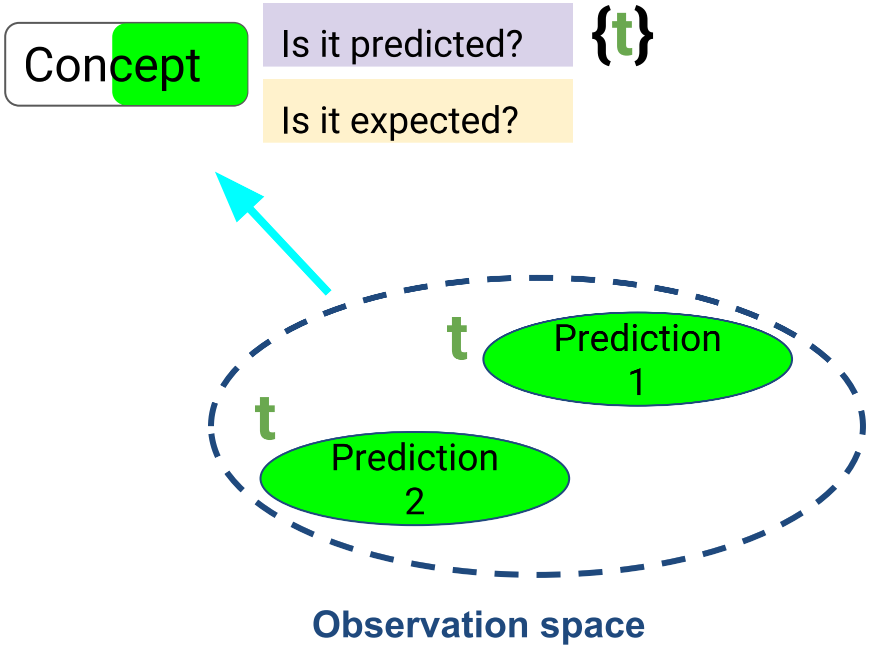

# Observations to concepts

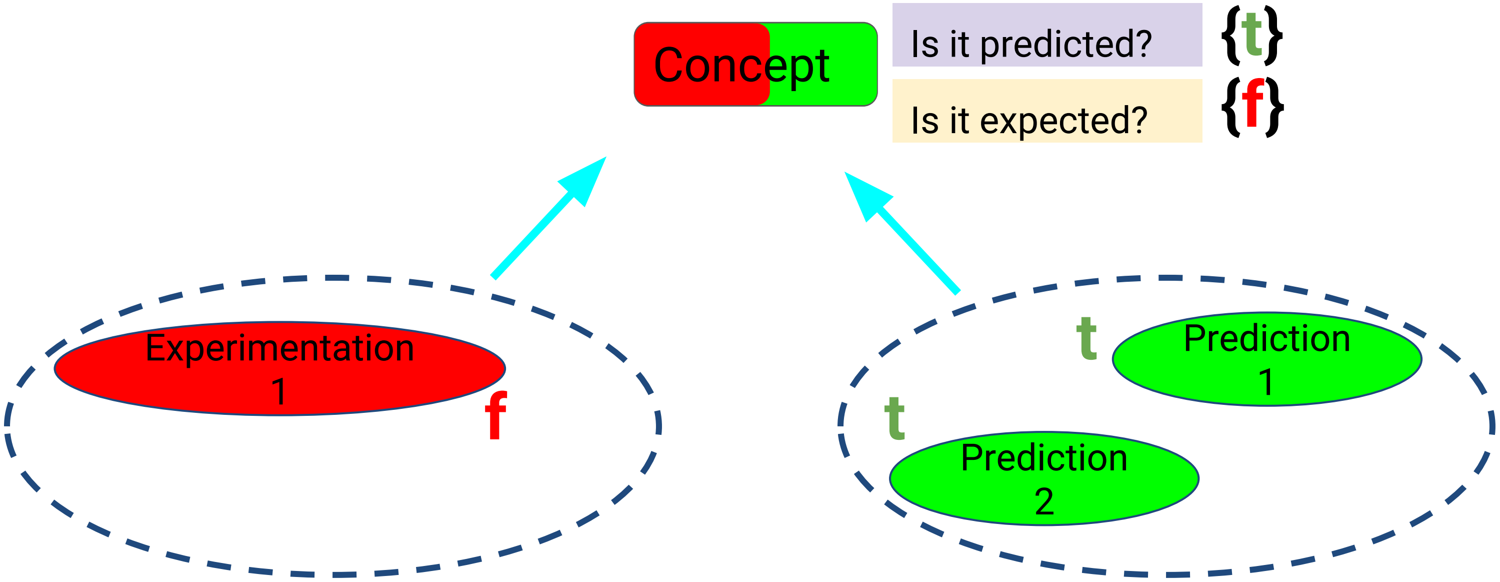

# Observations to concepts

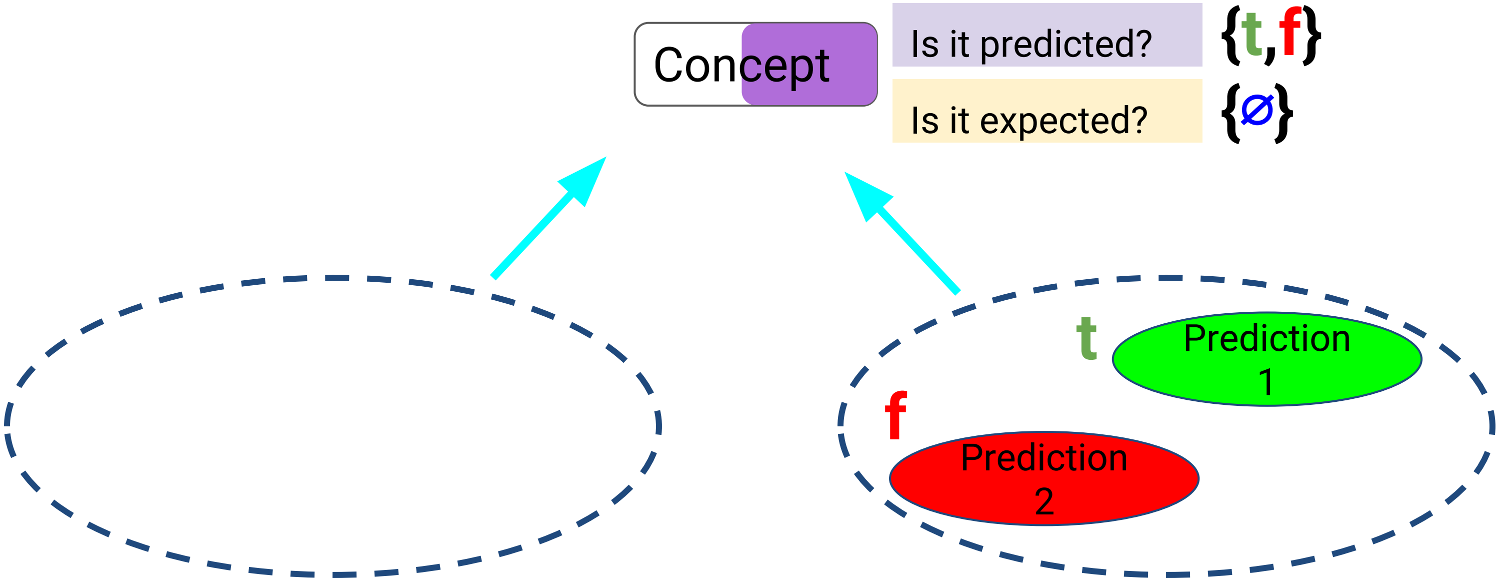

# Propagation rules

**4 rules**:  to propagate **predictions** or **expectations** following relation types (**composition** or **generalization/specialisation**)

# Propagation rules

**4 rules**:  to propagate **predictions** or **expectations** following relation types (**composition** or **generalization/specialisation**)

Propagation rule for predictions with composition relations

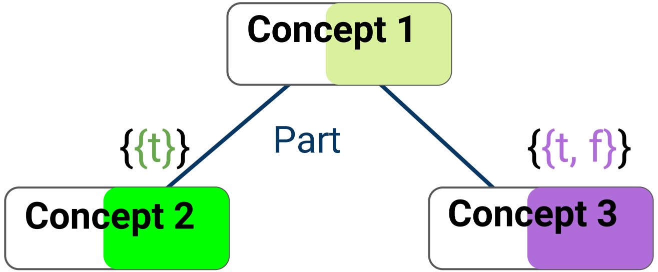

# Propagation rules

**4 rules**: to propagate **predictions** or **expectations** following relation types (**composition** or **generalization/specialisation**)

Propagation rule for predictions with composition relations

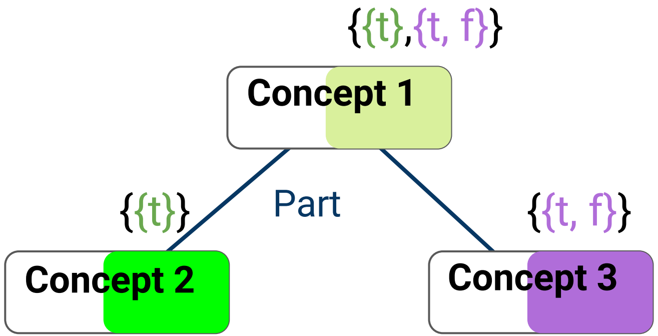

# Propagation rules

**4 rules**: to propagate **predictions** or **expectations** following relation types (**composition** or **generalization/specialisation**)

Propagation rule for predictions with generalization/specialisation relations

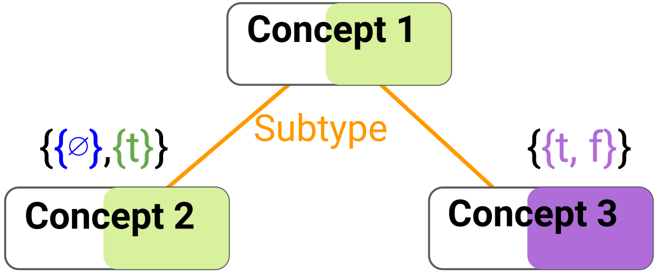

# Propagation rules

**4 rules**:  to propagate **predictions** or **expectations** following relation types (**composition** or **generalization/specialisation**)

Propagation rule for predictions with generalization/specialisation relations

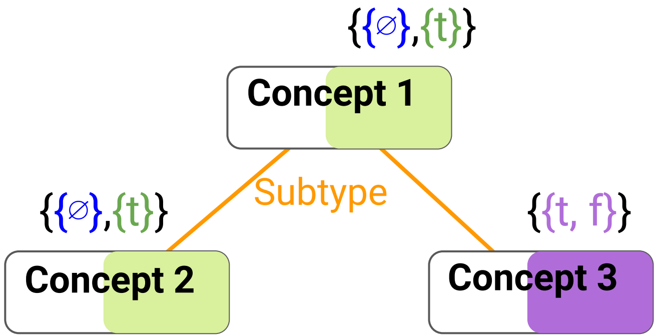

# Reasoning: specific mode

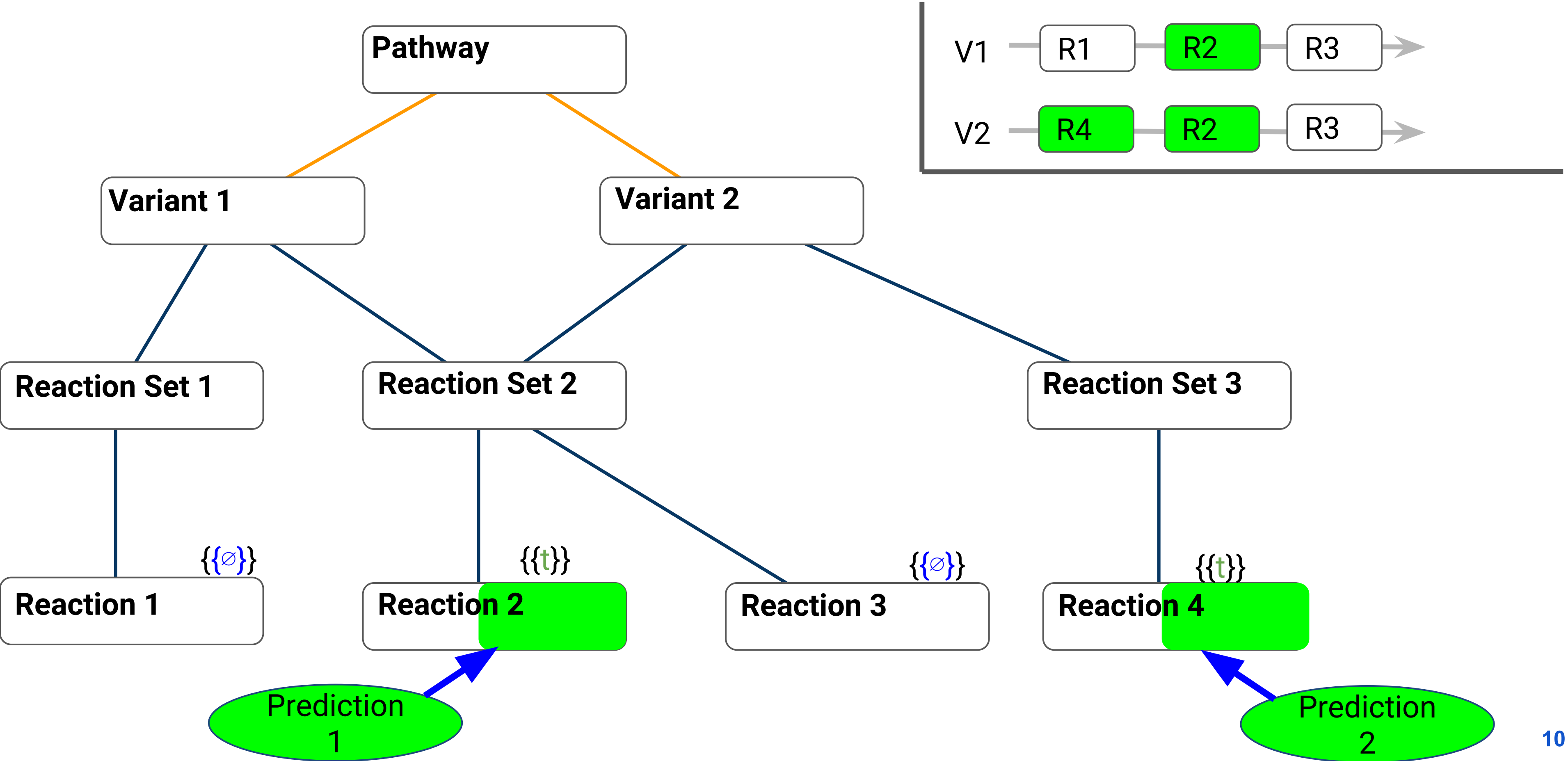

# Reasoning: specific mode

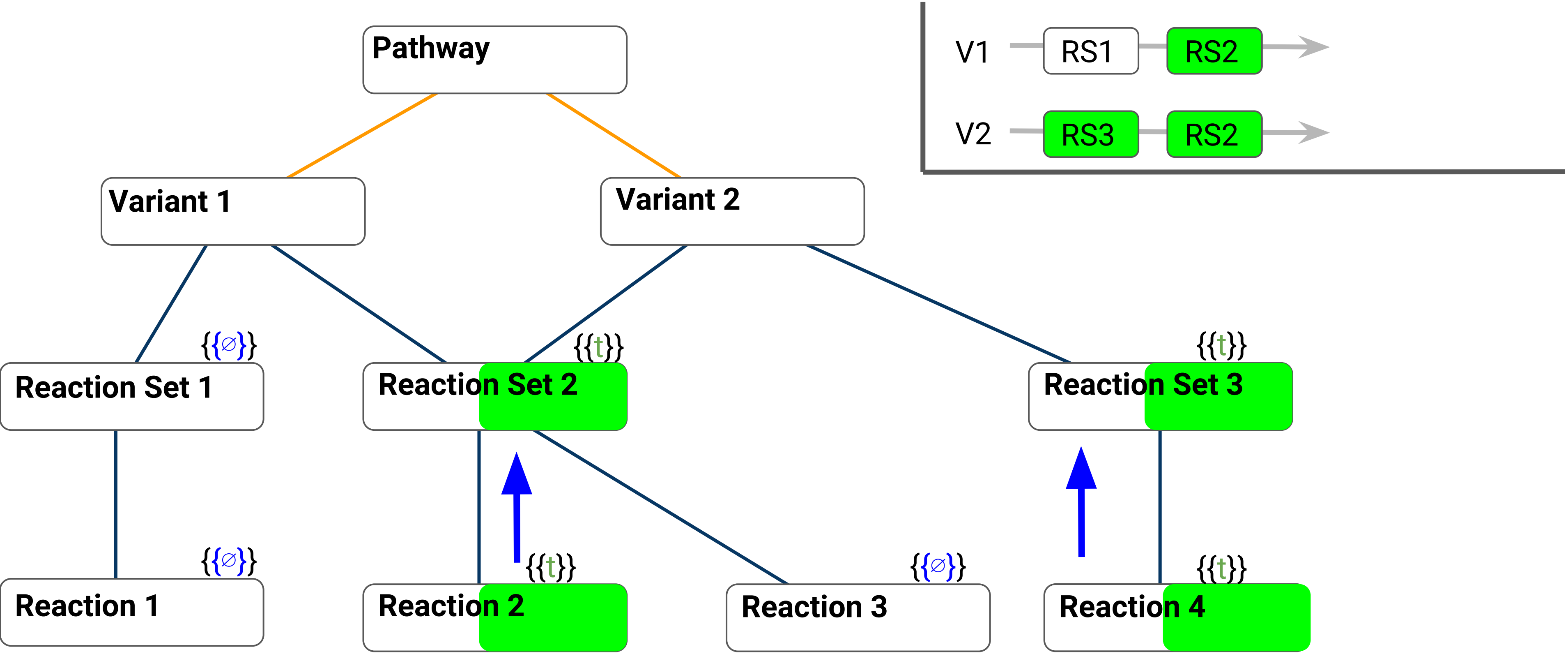

# Reasoning: specific mode

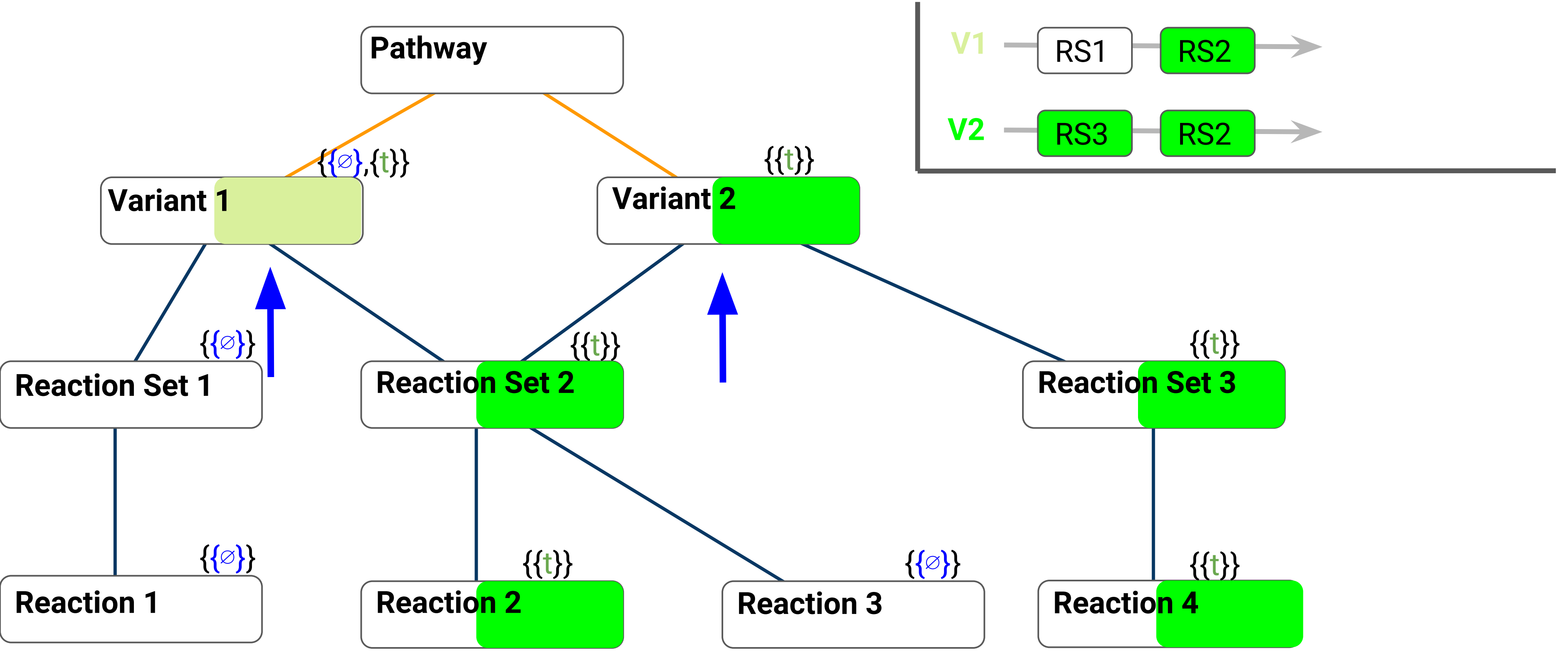

# Reasoning: specific mode

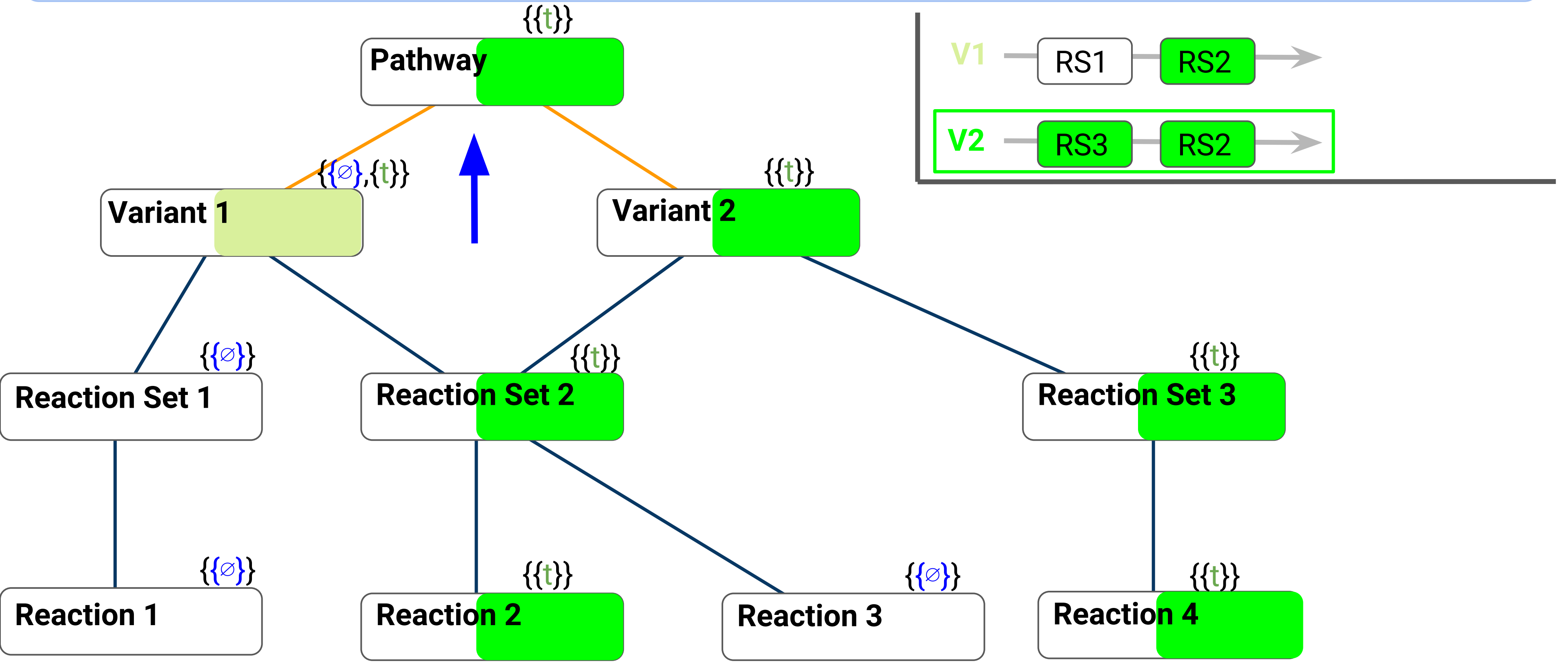

# Reasoning: specific mode

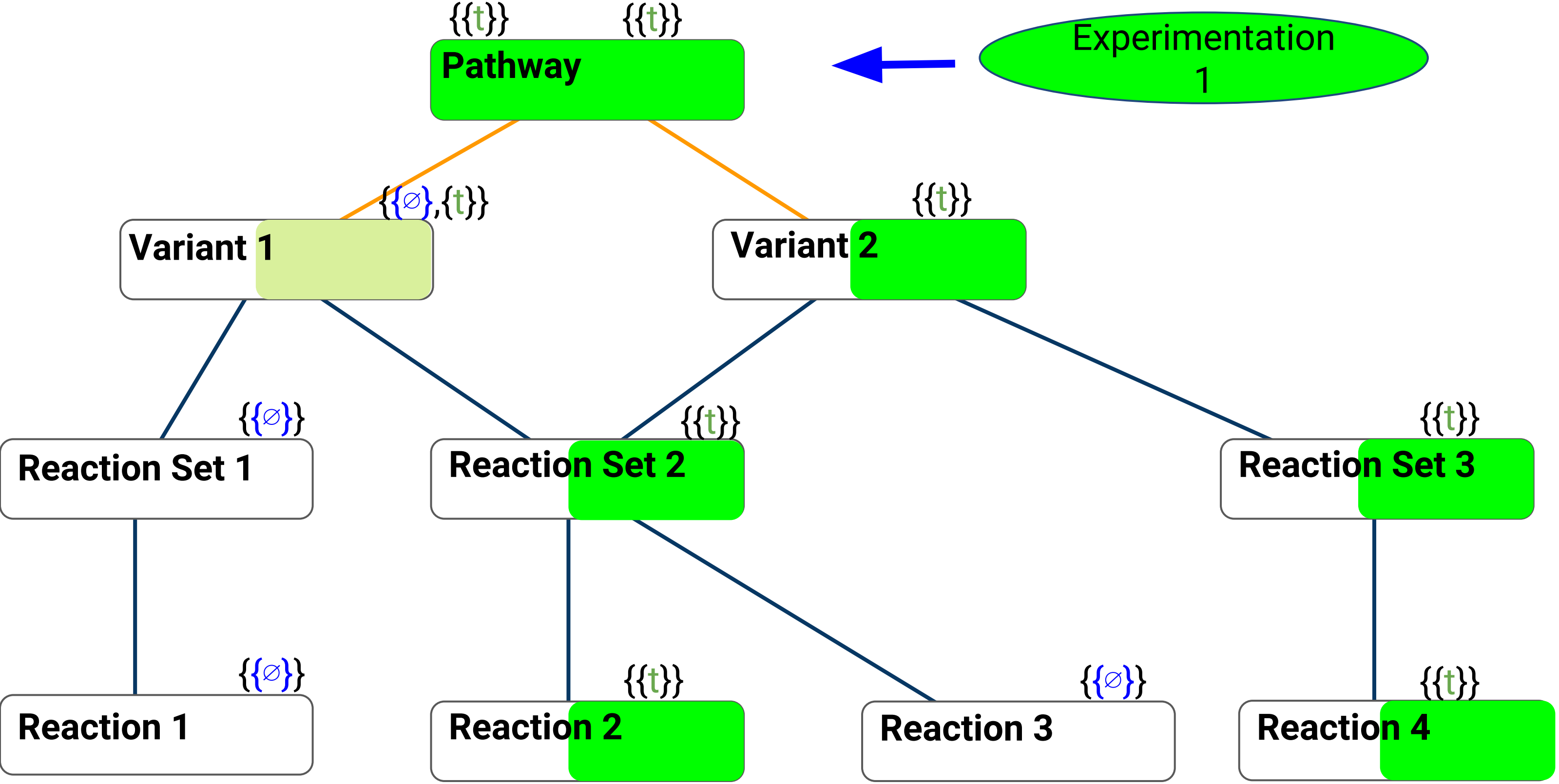

# Reasoning: specific mode

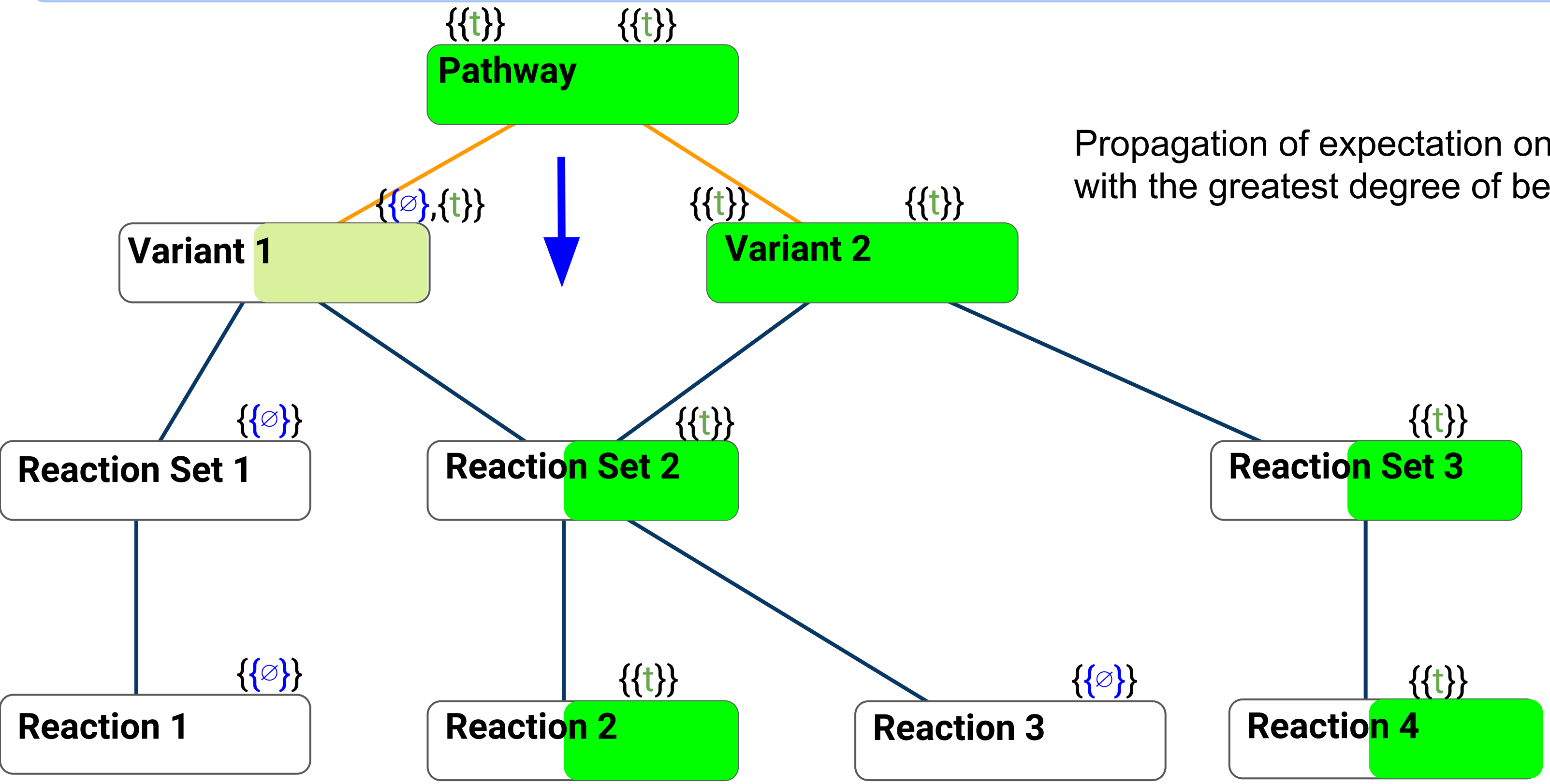


Propagation of expectation on child concept with the greatest degree of belief for prediction

# Reasoning: specific mode

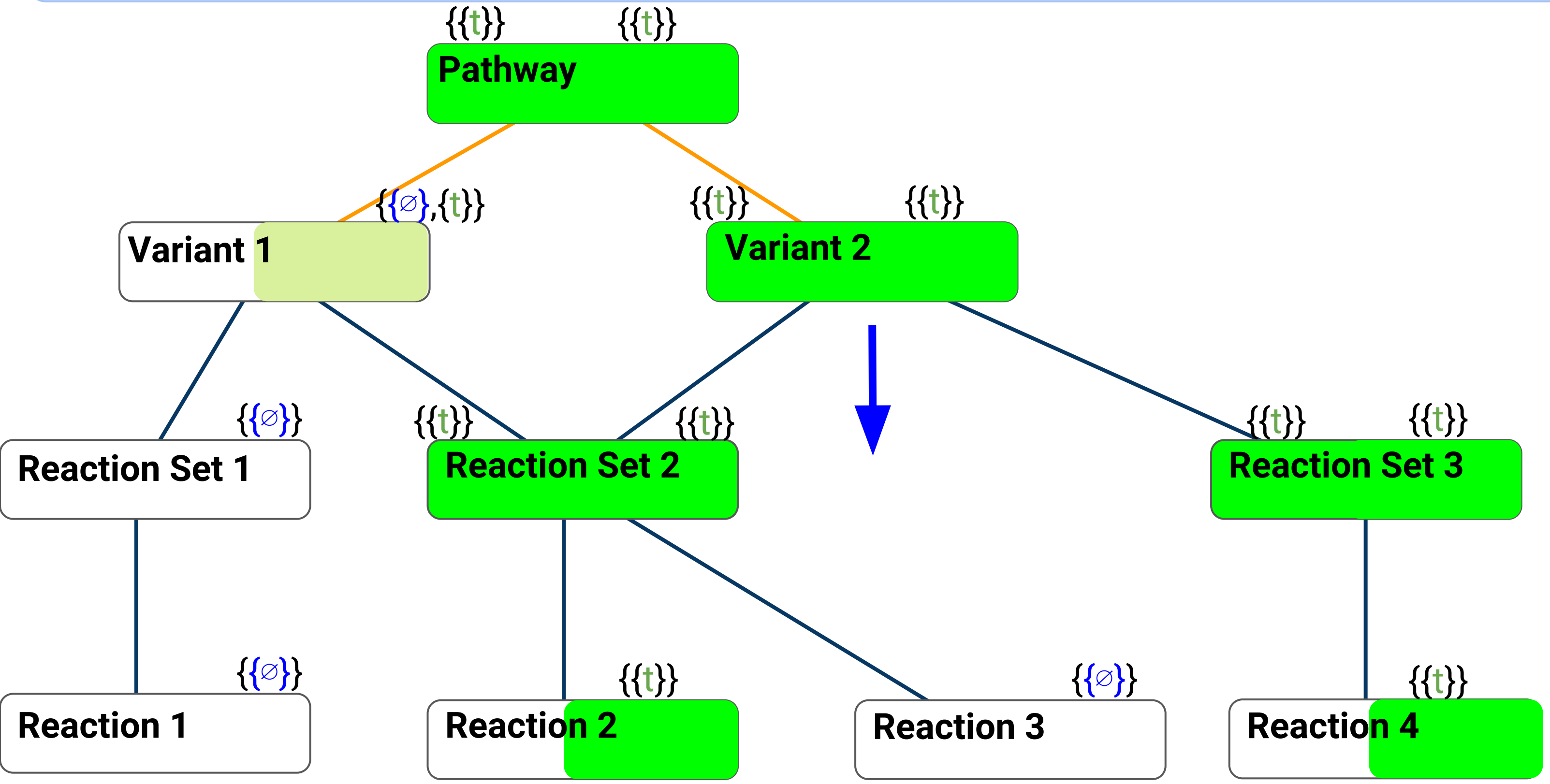

# Reasoning: specific mode

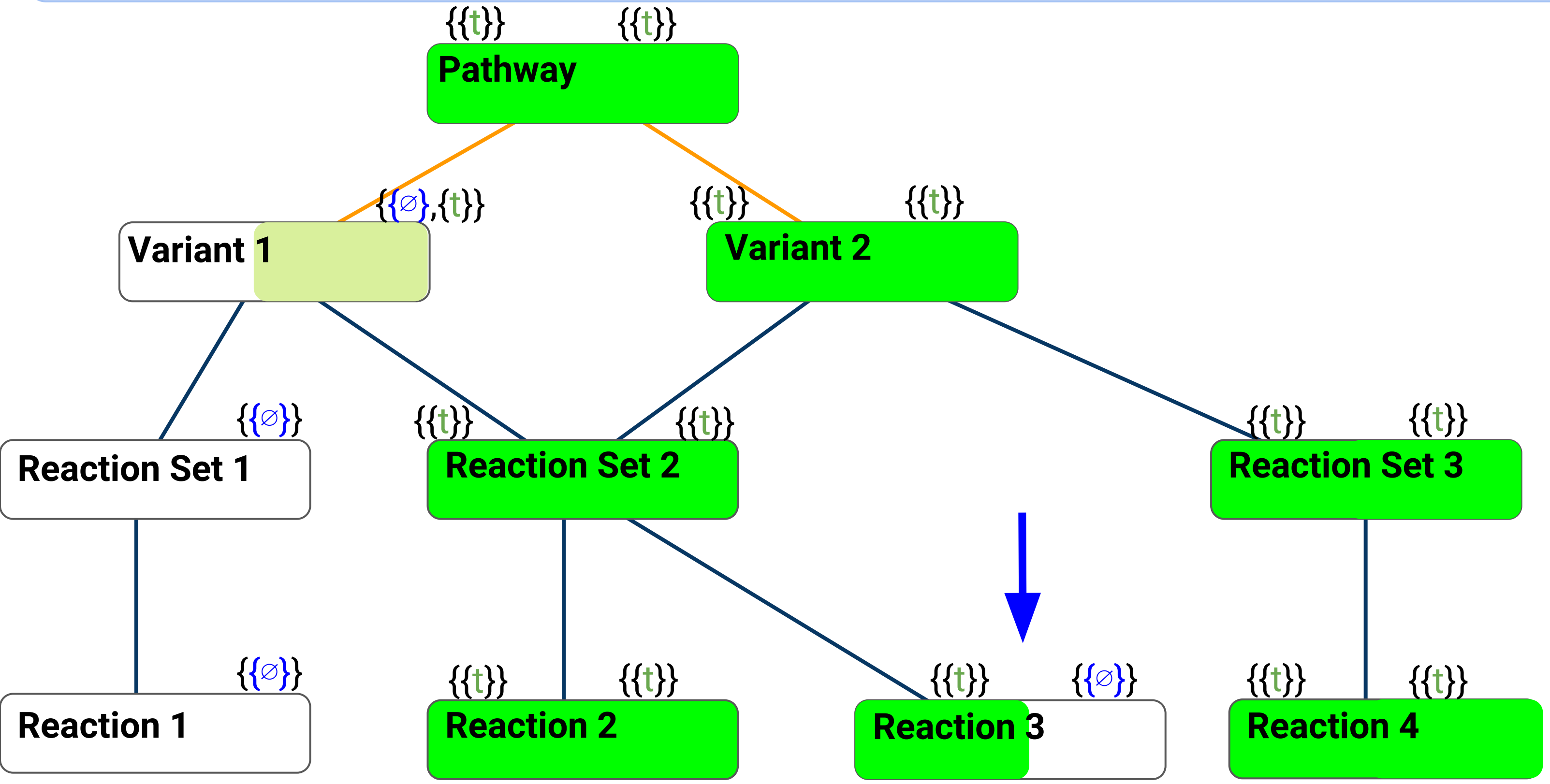

# Reasoning: conclusions

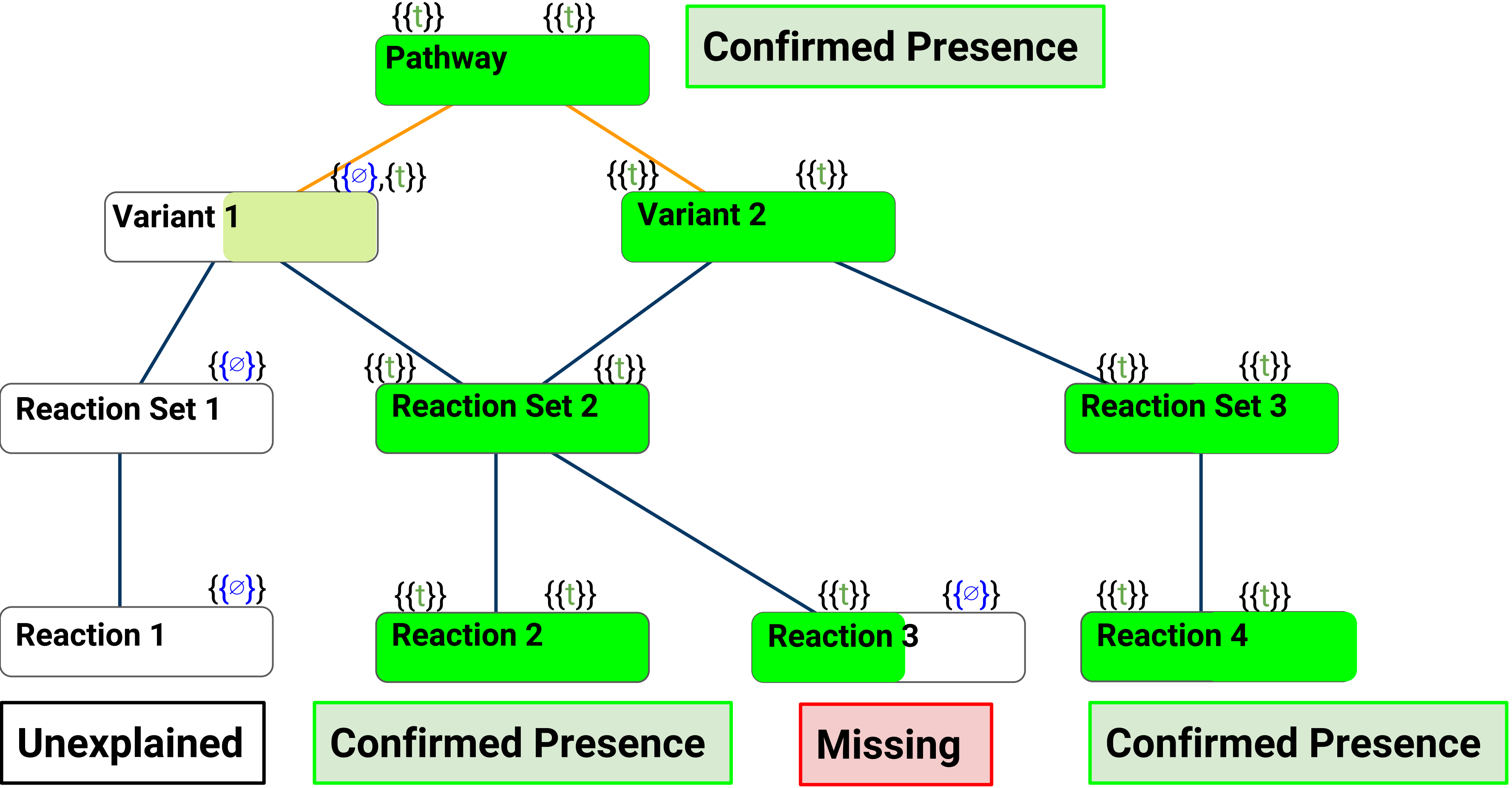

Supplement: Supplementary file 1 — Graphical illustration of GROOLS reasoning. (PDF 143 kb) [file 12859_2018_2126_MOESM1_ESM.pdf]
