# Supplementary material for: GROOLS: reactive graph reasoning for genome annotation through biological processes
Source: BMC Bioinformatics. 2018 Apr 11;19:132. doi: 10.1186/s12859-018-2126-1 (PMC5896057; doi:10.1186/s12859-018-2126-1)
Supplement: Supplementary file 2 — Accuracy and F1 scores with detailed counts. (PDF 55 kb) [file 12859_2018_2126_MOESM2_ESM.pdf]

Table 1: Prediction accuracy and F1 scores for pathways (PWY) and functional units (FU) in 14 organisms

Organisms (UniProtKB proteome)		Genome Properties: UniProtKB annotations, falsehood mode						UniPathway: MicroScope annotaions, specific mode					
		TP	TN	FP	FN	ACC	F1 score	TP	TN	FP	FN	ACC	F1 score
Acinetobacter baylyi ADP1 (UP000000430)	PWY	13	8	4	2	77.78%	81.25%	21	10	5	3	79.49%	84.00%
	FU	75	104	14	3	91.33%	89.82%	272	35	0	7	97.77%	98.73%
Pseudomonas putida KT2440 (UP000000556)	PWY	17	6	4	2	79.31%	85.00%	28	9	5	10	71.15%	78.87%
	FU	96	55	6	3	94.38%	95.52%	222	35	2	19	92.45%	95.48%
Escherichia coli K-12 (UP000000625)	PWY	18	4	5	1	78.57%	85.71%	27	6	2	9	75.00%	83.08%
	FU	138	47	10	1	94.39%	96.17%	111	17	5	15	86.49%	91.74%
Delftia acidovorans DSM14801 (UP000000784)	PWY	16	6	3	2	81.48%	86.49%	27	10	4	5	80.43%	85.71%
	FU	105	35	3	7	93.33%	95.45%	96	34	7	25	80.25%	85.71%
Agrobacterium tumefaciens C58 (UP000000813)	PWY	17	3	3	6	68.97%	79.07%	25	2	2	12	65.85%	78.13%
	FU	100	25	6	56	66.84%	76.34%	146	3	2	37	79.26%	88.22%
Pedobacter heparinus DSM2366 (UP000000852)	PWY	8	7	1	9	60.00%	61.54%	18	12	8	6	68.18%	72.00%
	FU	62	51	2	103	51.83%	54.15%	68	51	19	32	70.00%	72.73%
Roseobacter litoralis DSM6996 (UP000001353)	PWY	11	3	3	4	66.67%	75.86%	20	4	3	1	85.71%	90.91%
	FU	69	29	3	10	88.29%	91.39%	77	9	6	16	79.63%	87.50%
Bacillus subtilis 168 (UP000001570)	PWY	18	4	3	1	84.62%	90.00%	23	6	5	4	76.32%	83.64%
	FU	130	33	10	1	93.68%	95.94%	296	26	0	18	94.71%	97.05%
Sphingomonas wittichii DSM6014 (UP000001989)	PWY	12	4	6	5	59.26%	68.57%	22	11	9	5	70.21%	75.86%
	FU	85	94	9	12	89.50%	89.01%	87	59	14	12	84.88%	87.00%
Spirosoma linguale DSM74 (UP000002028)	PWY	10	6	4	8	57.14%	62.50%	18	11	5	4	76.32%	80.00%
	FU	64	54	6	84	56.73%	58.72%	66	36	9	12	82.93%	86.27%
Chitinophaga pinensis DSM2588 (UP000002215)	PWY	9	4	4	8	52.00%	60.00%	18	12	10	5	66.67%	70.59%
	FU	62	40	15	49	61.45%	65.96%	69	51	26	28	68.97%	71.88%
Pseudomonas aeruginosa PAO1 (UP000002438)	PWY	18	3	3	4	75.00%	83.72%	27	8	3	5	81.40%	87.10%
	FU	130	29	3	5	95.21%	97.01%	108	27	5	17	85.99%	90.76%
Kytococcus sedentarius DSM20547 (UP000006666)	PWY	3	5	5	12	32.00%	26.09%	15	16	7	9	65.96%	65.22%
	FU	39	90	14	61	63.24%	50.98%	47	69	19	44	64.80%	59.87%
Salmonella typhimurium SL1344 (UP000008962)	PWY	14	3	5	1	73.91%	82.35%	21	13	9	2	75.56%	79.25%
	FU	81	24	16	1	86.07%	90.50%	77	52	26	1	82.69%	85.08%
