# Supplementary material for: GROOLS: reactive graph reasoning for genome annotation through biological processes
Source: BMC Bioinformatics. 2018 Apr 11;19:132. doi: 10.1186/s12859-018-2126-1 (PMC5896057; doi:10.1186/s12859-018-2126-1)
Supplement: Supplementary file 3 — Textual descriptions for both examples illustrated in Figs. 3 and 4. (PDF 78 kb) [file 12859_2018_2126_MOESM3_ESM.pdf]

GROOLS Prior Knowledge descriptions for both examples illustrated in figures 3 and 4

Table of Figure 3

Prior Knowledge	Description	Expectation	Prediction	Conclusion
GenProp0305	cysteine biosynthesis~The biosynthesis of the amino acid cysteine may be achieved via several routes. Most commonly serine is the starting point (GenProp0218), while in the archaea a mis-charged cys-tRNA bearing O-phosphoserine is modified (GenProp0304).	True - {{t}}	Both - {{t},{f}}	Contradictory absence
Evidence_85853	GenProp0304 GENPROP	None - {{∅}}	False - {{f}}	Unconfirmed absence
Component_48840	serine O-acetyltransferase	True - {{t}}	False - {{f}}	Unexpected absence
Component_64077	cysteine biosynthesis	True - {{t}}	Both - {{t},{f}}	Contradictory absence
Evidence_61338	TIGR01138 HMM	None - {{∅}}	False - {{f}}	Unconfirmed absence
Component_51214	O-phosphoseryl-tRNA synthetase	None - {{∅}}	False - {{f}}	Unconfirmed absence
Evidence_85852	GenProp0218 GENPROP	True - {{t}}	Both - {{t},{f}}	Contradictory absence
Component_48841	cysteine synthase	True - {{t}}	True - {{t}}	Confirmed presence
GenProp0218	cysteine biosynthesis from serine~The conversion of L-serine to L-cysteine takes two enzymatic steps, described here. The first step involves Serine acetyl-transferase, and the second step involves acetylserine lyase which forms L-cysteine.	True - {{t}}	Both - {{t},{f}}	Contradictory absence
Evidence_65816	TIGR02539 HMM	None - {{∅}}	False - {{f}}	Unconfirmed absence
Component_51215	Sep-tRNA:Cys-tRNA synthase	None - {{∅}}	False - {{f}}	Unconfirmed absence
Evidence_61340	TIGR01172 HMM	True - {{t}}	False - {{f}}	Unexpected absence
GenProp0304	cysteine biosynthesis, tRNA-dependent~Methanocaldococcus jannaschii is a model archaeal species that lacks a direct cys--tRNA ligase. In a system analogous to pyrrolysine and (in many species) asparagine tRNA aminoacylation, the tRNA is misacylated and then the attached amino acid is modified. This two-step pathway is conducted by proteins in the families of MJ1660 (sepS) and MJ1678 (pscS). This pathway assumes 0-phosphoserine, whose biosynthesis is proposed to require D-3-phosphoglycerate dehydrogenase (TIGR01327) and an unidentified aminotransferase.	None - {{∅}}	False - {{f}}	Unconfirmed absence
Evidence_61339	TIGR01139 HMM	True - {{t}}	True - {{t}}	Confirmed presence
Evidence_65815	TIGR00470 HMM	None - {{∅}}	False - {{f}}	Unconfirmed absence

Table of Figure 4A

Prior Knowledge	Description	Expectation	Prediction	Conclusion
UER00195	L-asparagine from L-aspartate (L-Gln route): step 1/1~1 ATP + 1 H(2)O + 1 L-aspartate + 1 L-glutamine => 1 AMP + 1 L-asparagine + 1 L-glutamate + 1 diphosphate.	True - {{t}}	None - {{∅}}	Missing
UER00194	L-asparagine from L-aspartate (ammonia route): step 1/1~1 ATP + 1 L-aspartate + 1 NH(3) => 1 AMP + 1 L-asparagine + 1 diphosphate.	True - {{t}}	None - {{∅}}	Missing
ULS00080	L-asparagine from L-aspartate (ammonia route)	True - {{t}}	None - {{∅}}	Missing
UPA00134	L-asparagine biosynthesis~Biosynthesis of L-asparagine amino-acid.	True - {{t}}	None - {{∅}}	Missing
ULS00081	L-asparagine from L-aspartate (L-Gln route)	True - {{t}}	None - {{∅}}	Missing
VariantPath-1-UPA00134		True - {{t}}	None - {{∅}}	Missing
VariantPath-2-UPA00134		True - {{t}}	None - {{∅}}	Missing

Table of Figure 4B

Prior Knowledge	Description	Expectation	Prediction	Conclusion
UER01034	L-aspartyl-[tRNAAsn] from L-aspartate~a tRNAAsx + L-aspartate + ATP => an L-aspartyl-[tRNAAsx] + AMP + diphosphate	True - {{t}}	True - {{t}}	Confirmed presence
VariantPath-3-UPA00134		True - {{t}}	True - {{t}}	Confirmed presence
UER01035	L-asparaginyl-[tRNAasn] from L-aspartyl-[tRNAAsn]~L-glutamine + an L-aspartyl-[tRNAAsn] + ATP + H2O => L-glutamate + an L-asparaginyl-[tRNAasn] + ADP + phosphate + H+	True - {{t}}	None - {{∅}}	Missing
UER00194	L-asparagine from L-aspartate (ammonia route): step 1/1~1 ATP + 1 L-aspartate + 1 NH(3) => 1 AMP + 1 L-asparagine + 1 diphosphate.	None - {{∅}}	None - {{∅}}	Unexplained
ULS00080	L-asparagine from L-aspartate (ammonia route)	None - {{∅}}	None - {{∅}}	Unexplained
UPA00134	L-asparagine biosynthesis~Biosynthesis of L-asparagine amino-acid.	True - {{t}}	True - {{t}}	Confirmed presence
UER01036	L-asparagine from L-asparaginyl-[tRNAasn]~an L-asparaginyl-[tRNAasn] + H20 => L-asparagine + tRNAasn	True - {{t}}	True - {{t}}	Confirmed presence
ULS00081	L-asparagine from L-aspartate (L-Gln route)	None - {{∅}}	None - {{∅}}	Unexplained
VariantPath-2-UPA00134		None - {{∅}}	None - {{∅}}	Unexplained
ULS00511	L-asparagine from L-aspartate (tRNA-dependent route)	True - {{t}}	True - {{t}}	Confirmed presence
VariantPath-1-UPA00134		None - {{∅}}	None - {{∅}}	Unexplained
UER00195	L-asparagine from L-aspartate (L-Gln route): step 1/1~1 ATP + 1 H(2)O + 1 L-aspartate + 1 L-glutamine => 1 AMP + 1 L-asparagine + 1 L-glutamate + 1 diphosphate.	None - {{∅}}	None - {{∅}}	Unexplained
